# Investigating nutrient intake during use of glucagon-like peptide-1 receptor agonist: a cross-sectional study

**DOI:** 10.3389/fnut.2025.1566498

**Published:** 2025-04-25

**Authors:** Brittany Johnson, Mary Milstead, Olena Thomas, Tyler McGlasson, Lauren Green, Rachel Kreider, Rachel Jones

**Affiliations:** GNC Holdings, LLC, Pittsburgh, PA, United States

**Keywords:** weight loss, nutrient intake, glucagon-like peptide-1 receptor agonists, nutrition, diet, dietary reference intakes

## Abstract

**Background:**

Glucagon-like peptide-1 receptor agonist (GLP-1RA) pharmaceutical interventions have advanced medical treatment for obesity, yet little is known about nutrient intake while using a GLP-1RA. The purpose of this study was to compare nutrient intake while using a GLP-1RA to the Dietary Reference Intakes (DRI).

**Methods:**

A cross-sectional study was conducted in a sample of participants who had been using GLP-1RA for at least one month (*N* = 69). Participants answered online survey questionnaires and completed a 3-day food record. Descriptive statistics (means, standard deviations) were calculated for all participant demographic characteristics and average 3-day nutrient intakes. Average 3-day nutrient intakes were compared to the DRI using 95% confidence intervals (CI). A Bonferroni correction applied accepted significance at *p* ≤ 0.00156. One-way ANOVA analysis was conducted to compare the self-reported MyPlate servings to recorded servings from the 3-day food record.

**Results:**

Compared to the DRI reference values, participants consumed adequate amounts of B-vitamins, copper, phosphorus, selenium, and zinc. Participants had insufficient intakes of several key nutrients below the DRI, including fiber (14.5 g; CI: 12–17), calcium (863 mg; CI: 756–970), iron (12.1 mg; CI: 11–13), magnesium (266 mg; CI: 236–297), potassium (2,186 mg; CI: 1,969–2,402), choline (305 mg; CI: 268–342), vitamin A (560 mcg RAE; CI: 469–651), vitamin C (51 mg; CI: 41–61), vitamin D (4 mcg; CI: 3–5), vitamin E (9.6 mg; CI: 8–11), and *p* < 0.00156. Participants overconsumed % calories from fat (39.9%; CI: 38, 42), and saturated fat (26 g; CI: 26, 26), *p* < 0.00156. Participants did not meet the daily recommended MyPlate servings for fruit, vegetables, grains, or dairy (*p* < 0.01). Protein intake (% total calories) was within the AMDR, however based on a g/kg/day, protein intake was significantly under daily needs.

**Conclusion:**

Participants on a GLP-1RA are not meeting the DRI for several vital nutrients through their diet or higher protein needs during weight loss. Patient-centered nutritional guidance is essential to optimize health outcomes and prevent unintended health consequences. Future large-scale studies are needed to assess the replicability of these findings and provide custom nutritional guidance for those on a GLP-1RA medication.

## Graphical abstract

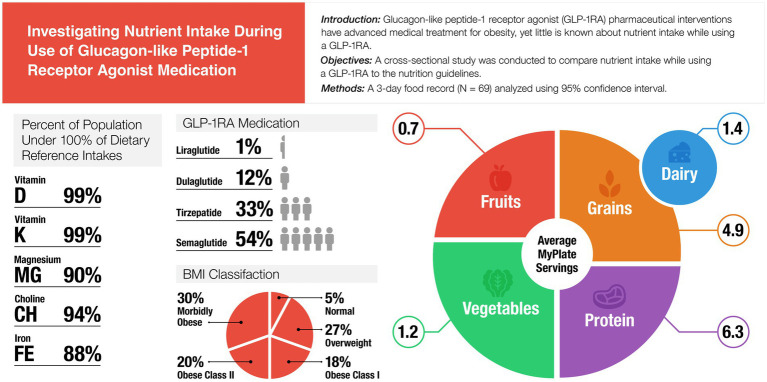

## 1 Introduction

Since 1990, the prevalence of obesity has doubled with one in eight people globally and one in five adults in the United States living with obesity (1, 2). Recent pharmaceutical interventions, specifically glucagon-like peptide-1 receptor agonist drugs (GLP-1RA), have advanced medical treatment for obesity and glycemic control (3). Despite the proven weight loss efficacy of GLP-1RA, adequate attention to addressing nutritional concerns has been lacking. GLP-1RA slows the transit time of the gastrointestinal tract and suppresses appetite, which has the potential to displace nutrient intake and lead to vitamin and mineral deficiencies (4). Further, side effects, such as nausea and vomiting can interfere with food choices, leading to suboptimal intakes of certain food groups. Achieving optimal health outcomes with GLP-1RA therapy requires a multifaceted approach that extends beyond just pharmacological intervention. Lifestyle modifications, such as exercise and dietary habits, are essential complementary behavior changes to promote optimal health while taking a GLP-1RA. Therefore, it is critical to address nutrient status to optimize outcomes for GLP-1RA patients.

Patient-centered care from a holistic, evidence-based approach is crucial to ensure safe and sustainable weight loss. Little to no medical nutrition therapy (MNT) guidelines are provided to health care providers or patients on adequate nutrient intake while using GLP-1RA based on data from clinical trials in this population. There is a growing call to action for more integrated care plans that combine GLP-1RA with targeted diet and life-style modifications to enhance patient outcomes. A report from the Academy of Nutrition and Dietetics emphasizes the crucial role of Registered Dietitian Nutritionists (RDN) in providing comprehensive management of obesity (5). Practicing RDNs have highlighted a lack of quality nutrition education for GLP-1RA patients, noting similarities to the early stages of bariatric surgery when comprehensive dietary guidance was also insufficient (6). Current clinical guidance focuses primarily on managing gastrointestinal side effects for GLP-1RA patients, with some nutrition considerations recommendations based on previous literature in weight loss science (7–10). Significant gaps remain with nutrition interventions while using a GLP-1RA related to optimal nutrient intake and timing. Traditional dietary weight loss interventions primarily emphasize calorie restriction with MNT protocols focusing on consuming nutrient-dense foods. The use of GLP-1RA is a rapidly advancing therapy for weight loss, garnering the need to create a tailored MNT protocol, similar to bariatric-specific MNT.

Beyond nutrient recommendations, considerations for changes in body composition to prevent loss of muscle mass are needed, as both are critical for weight management and metabolic health post-GLP-1RA (11). A once-weekly GLP-1RA injection provides approximately 14.9% weight loss compared to an average 5 to 7% using traditional diet and lifestyle modifications (12, 13). This rapid weight loss leads to significant changes in body composition, most concerning the loss of muscle mass. Research documents GLP-1RA users lose around 20–50% of lean body mass, which is much higher than traditional weight loss by diet and exercise (11). Several clinical trials have found consuming more protein than the Recommended Dietary Allowance (RDA) not only reduces body weight but also enhances body composition. The established RDA of 0.8 g/kg/day was intended for minimum daily protein needs for general populations. However, during weight loss higher protein needs are necessary to maintain a positive protein balance. A positive protein balance decreases fat mass and preserves lean mass in both low-calorie and standard-calorie diets (14–16). To help preserve lean mass during hypocaloric diets, 1.2–2.0 g/kg of protein should be consumed daily (10). Long-term weight management is highly correlated with the retention of lean mass during weight loss. It is critical to address the importance of higher protein needs for this population, as the DRI levels are likely insufficient to meet higher demands to support lean mass.

Another area lacking empirical guidance is recommendations on specific nutrient needs or food groups for GLP-1RA patients. Adequate nutrient intake is essential to promote health, reduce chronic disease, and prevent deficiencies and toxicities (17). Therefore, expert panels and committees from the Food and Nutrition Board of the Institute of Medicine developed the Dietary Reference Intakes (DRI). The DRIs influence public policy and guide nutritional recommendations for general populations based on calories, males/females and age to promote health and prevent chronic disease. The DRIs play a critical role in shaping nutrition recommendations, including the Dietary Guidelines for Americans (DGA) and MyPlate (18). MyPlate is visual tool providing dietary guidance for Americans on recommended daily food group servings. However, there are currently no specific DRIs or MyPlate guidance for low-calorie diets or medical weight loss. These gaps in knowledge limit our understanding of specific nutrients and food group requirements while using a GLP-1RA. Reduced calorie intake is correlated with lower vitamin and mineral consumption, but there is limited data available addressing micronutrient requirements during calorie restriction (19). Publications across the landscape of obesity interventions can provide some evidence for the nutritional status and insights for the GLP-1RA population. For example, a previous study measuring baseline nutritional status of individuals with obesity found prevalence of micronutrient deficiencies from serum concentration for vitamin D, vitamin C, selenium, and iron (20). Another study for micronutrient status prior to bariatric surgery found 48.7% of individuals with morbid obesity showed at least one prevalent deficiency in key serum nutrients but vitamin and mineral intake from food was not recorded (21). The nutritional status of individuals taking GLP-1RA plays a pivotal role in treatment effectiveness and overall health outcomes. Due to reduced food intake from GLP-1RA, it’s hypothesized nutrient deficiencies are prevalent while taking GLP-1RA. A handful of clinical studies have investigated food intake while using a GLP-1RA. In a 14-week clinical trial using three 24-h food recalls, participants using 1.8 mg/d liraglutide injection reduced energy intake on average by 294 calories over the study period (22). Another study evaluated ad libitum intake during week 12 of Semaglutide treatment and found a 24% reduction in total calories compared to placebo for the one-day energy intake measured in a feeding lab (23). Gibbons et al. found a 38.9% lower total energy intake compared to placebo during week 12 of oral semaglutide treatment (24). However, none of these studies reported specific micronutrient intake. Additionally, there is limited data on the overall food servings consumed by individuals using a GLP-1RA.

To our knowledge, no study has investigated dietary intake during GLP-1RA treatment and analyzed nutrient intakes compared to DRI. Given the importance of adequate nutritional status to promote health and prevent chronic disease, insights into the dietary patterns and nutritional needs of this population are imperative to inform evidence-based clinical guidance and optimize therapeutic outcomes. Identifying common nutrient deficiencies associated with GLP-1RA use can guide targeted nutritional interventions to enhance treatment and mitigate adverse effects. As GLP-1RA becomes increasingly popular with little evidence about long-term deficiencies or sustainable weight loss, investigations in nutritional science are an important aspect to best support the outcomes for these individuals. Thus, the purpose of this study was to compare the nutrient intakes of individuals using a GLP-1RA for weight loss to the DRI. We hypothesize individuals using a GLP-1RA do not meet the daily nutrient needs.

## 2 Materials and methods

A cross-sectional study was completed with the population of interest which included current GLP-1RA users in the United States (U.S.). A convenience sample of eligible individuals was identified through an online research platform based on predefined inclusion and exclusion criteria and enrolled in this study. This approach streamlined the recruitment process and ensured access to a diverse and representative population for comprehensive data collection. Inclusion criteria to participate were: (1) currently using a GLP-1RA for at least one month, (2) willing to complete online survey questionnaires regarding their dietary intake and health habits, (3) willing to provide a detailed 3-day electronic food record, and (4) be at least 18 years of age. Participants were excluded if they were: (1) concurrently enrolled in a nutrition program or (2) receiving meal plans. The research procedures were approved by an Institutional Review Board and all participants provided informed consent prior to voluntary participation (BRANY IRB-Manager, New York, United States; Approval date 16 August 2024). Recruitment occurred September 2024 to October 2024 and consisted of online invitations identifying qualified participants. Individuals who expressed interest completed an online consent form and completed the survey questionnaire. Upon completing the survey, an email invitation was sent to provide instructions to access a personal login for the Automated Self-Administered 24-Hour Dietary Assessment Tool (ASA24) to collect the 3-day food record (25). The estimated sample size for this study was based on previous research in a GLP-1RA population (22), assuming a moderate effect size (d = 0.05) for anticipating a large deviation from the DRI. A G*Power analysis using the variable of protein intake as a percentage of total calories with a standard deviation of 5.5, and power of 0.80 at *p* < 0.05, indicated a minimum of 34 participants was required.

### 2.1 Survey questionnaires

Self-reported questionnaires for demographics, GLP-1RA usage, anthropometrics, and diet habits were collected online. Participants were asked healthcare-related questions regarding the education they received from their healthcare provider when prescribed the medication, as well as their level of satisfaction with the information provided. Participants were asked questions about their use of GLP-1RA, including the specific medication they were taking, the reason for its use, duration of treatment, anticipated length of future use, and any experienced side effects. Participants reported general dietary habits, indicating whether their food choices had changed since starting a GLP-1RA, since there’s currently no validated questionnaire designed for this population. Before tracking their dietary intake, participants were asked to self-report estimates of their daily servings for each MyPlate food group. To help improve accuracy, examples for each food group were provided to help them understand the correct serving sizes. For example, one serving of fruit (1 cup equivalent) = 1 medium fruit or 1 cup of fresh fruit. There is currently no validated questionnaire for measuring subjective MyPlate servings, however we wanted to compare self-reported servings to actual servings recorded to better understand potential knowledge gaps. Therefore, the estimates reported in the questionnaire served as a baseline measure of perceived nutritional intake to compare with the food group servings recorded in the 3-day food record. For this study, the recommended daily servings were based on MyPlate recommendations for ages 14 and up at a 2000 calorie level. Additionally, participants self-reported their height (feet and inches), current weight (pounds), weight prior to starting GLP-1RA, and goal weight. The height reported in feet and inches was calculated to centimeters and the weight in pounds was calculated to kilograms. Body mass index (BMI) was calculated using self-reported height and both starting weight and current weight. While a measured weight would offer greater accuracy, the online format of the study limited this option.

### 2.2 Three-day food record

The three-day food record was selected as the primary method to obtain nutrient intake for its strengths, such as recording actual intakes rather than food group servings, as well as real-time data collection, which reduces recall bias such as a 24-h recall (26). While each dietary assessment data collection tool has limitations, a food record was selected to provide additional insights into dietary intake not previously studied in GLP-1RA. Three-day food records are deemed valid for average intakes when compared to longer durations and reduce food log fatigue. Similar intakes have been observed comparing three days to longer durations and deemed valid for averaging intakes and an acceptable dietary assessment tool (27). Therefore, participants were asked to complete a 3-day food record using the Automated Self-Administered Dietary Assessment Tool, version 2024 (ASA24) software developed by The National Cancer Institute in the United States for collecting detailed information about an individual’s dietary intake (25, 28). The ASA24 software was created to improve validity by collecting 24-recalls or food records electronically. Dietary assessments used in research have inherent strengths and limitations (26), however, the 3-day food record was selected to help identify key knowledge gaps related to dietary intake among patients using GLP-1RA (29). Participants were instructed to record their food intake for three consecutive days, starting on the first day of their weekly injection, to standardize dietary assessment across the GLP-1RA sample. Given the current lack of evidence on how GLP-1RA administration influences day to day food intake, this approach was selected to ensure consistency and minimize variability in the data collection. To our knowledge, it’s unknown how GLP-1RA effects dietary intake fluctuations throughout the week and the duration of treatment. Participants were provided with a protocol on how to record their food logs to improve accuracy. The protocol included training on system navigation, food selection process, portion size estimations, reviewing and editing food entries, and how to log more complex meals. The main variables analyzed included calories, macronutrients, vitamins and minerals, which can be found in Table 1 along with the units of measurements. Secondary variables analyzed were the MyPlate food groups found in Table 2 with their respective units of measurements and servings. The ASA24 is designed to improve the accuracy and efficiency of dietary assessments for research purposes. ASA24 was accessed and completed by participants online, allowing for convenient and user-friendly data collection. Participants recorded all food and beverages consumed over a 3-day period, including portion sizes, preparation methods, and brand names if available. ASA24 includes built-in features for data quality control, such as error checks and validations, visual examples of portion sizes, and probing to enhance the accuracy and reliability of dietary assessments and reduce bias (25). The 3-day nutrient analysis was calculated as an average intake for each participant. If participants recorded more than three days, any additional entries were excluded, and only the first three days of data were included in the analysis.

**Table 1:** Average three-day nutrient intake.

Variables	DRI	Daily intake		
		Mean	SD	95% CI Min, Max
Protein, g	50	77.3	29.2	70, 84**^‡^
Protein, %	10–35	18.5	6.0	17, 20**^‡^
Carbohydrates, g	275	184.6	91.1	163, 207**^‡^
Carbohydrates, %	45–65	41.5	10.2	39, 44**^‡^
Added Sugars, g	50	11.5	9.9	12, 12**^‡^
Fat, g	78	78.1	33.2	70, 86
Fat, %	20–35	39.9	7.8	38, 42**^‡^
Saturated fat, g	20	26.0	11.2	26, 26**^‡^
Linoleic acid, g	12^#^	15.4	8.3	13, 17*^‡^
Alpha-Linolenic acid, g	1.1^#^	1.5	1.0	1, 2**^‡^
Fiber, g	28	14.5	9.4	12, 17**^‡^
Calcium, mg	1,300	862.9	443.7	756, 970**^‡^
Copper, mg	0.9	1.0	0.6	0, 2**^‡^
Iron, mg	18	12.1	5.7	11, 13**^‡^
Magnesium, mg	420	266.2	127.1	236, 297**^‡^
Phosphorus, mg	1,250	1,260.8	478.2	1,146, 1,376
Selenium, mcg	55	104.4	42.0	94, 115**^‡^
Zinc, mg	11	10.1	4.5	9, 11
Potassium, mg	4,700	2,185.6	898.1	1,969, 2,402**^‡^
Sodium, mg	2,300	3,164.3	1,199.1	2,876, 3,453*^‡^
Vitamin A, RAE mcg	900	559.9	378.4	469, 651**^‡^
Vitamin C, mg	90	51.0	43.0	41, 61**^‡^
Vitamin D, mcg	20	4.0	2.6	3, 5**^‡^
Vitamin E, mg	15	9.6	6.6	8, 11**^‡^
Vitamin K, mg	120	101.1	70.9	84, 118*
Thiamin, mg	1.2	1.4	0.7	0, 3*
Riboflavin, mg	1.3	2.1	1.7	2, 3**^‡^
Niacin, mg	16	24.4	18.0	20, 29**^‡^
Vitamin B6, mg	1.7	2.0	2.4	1, 3
Folate, DFE mcg	400	401.7	271.6	336, 467
Vitamin B12, mcg	2.4	4.9	4.8	4, 6**^‡^
Choline, mg	550	304.6	153.9	268, 342**^‡^

DRI = Daily Reference Intakes; SD = Standard Deviation; 95% CI = 95% Confidence Interval. # = DRI for females; mL = milliliters; g = grams; mg = milligrams, mcg = micrograms; Protein (%), Carbohydrate (%), Fat (%) = % of total calories for each macronutrient; Daily intake = average three-day nutrient intake; * *p* < 0.05; **p < 0.001 represented for 95% CI; ^‡^ = Bonferroni-corrected *p*-value ≤ 0.00156.

**Table 2:** Comparison of self-reported servings and average three-day food record servings for MyPlate food groups.

Food group, rec.	Self-reported servings		Food record servings	*p*
Fruit, 2 servings (2 cups eq.)			**Group total** **0.7 ± 0.9 (95% CI:1,1)** ^‡^	0.000
	0 servings	5 (7.2%)	0.3 ± 0.4 (0.0–0.9)	0.024
	1–2 servings	52 (75.4%)	0.6 ± 0.7 (0.0–2.8)	
	3–4 servings	11 (15.9%)	1.2 ± 1.1 (0.1–2.9)	
	5+ servings	1 (1.4%)	1.9 ± 0 (n/a)	
Vegetables, 2.5 servings (2.5 cups eq.)			**Group Total** **1.2 ± 0.8 (95% CI:1,1)** ^‡^	0.000
	0 servings	8 (11.6%)	0.9 ± 0.7 (0.2–2.2)	0.01
	1–2 servings	42 (60.9%)	1.1 ± 0.8 (0.2–2.7)	
	3–4 servings	18 (26.1%)	1.6 ± 0.8 (0.5–3.8)	
	5+ servings	1 (1.4%)	3.0 ± 0 (n/a)	
Grains, 6 servings (6 oz. eq.)			**Group total** **4.9 ± 2.8 (95% CI:5,5)**^‡^	0.002
	0 servings	6 (8.7%)	2.98 ± 2.9 (0.0–6.9)	0.008
	1–2 servings	47 (68.1%)	4.7 ± 2.6 (0.5–13.1)	
	3–4 servings	15 (21.7%)	5.9 ± 2.3 (2.1–10.6)	
	5+ servings	1 (1.4%)	11.7 ± 0 (n/a)	
Dairy3 servings (3 cups eq.)			**Group total** **1.4 ± 0.8 (95% CI:1,1)**^‡^	0.000
	0 servings	5 (7.2%)	1.0 ± 0.8 (0.2–2.1)	0.342
	1–2 servings	48 (69.6%)	1.4 ± 0.9 (0.8–4.4)	
	3–4 servings	15 (21.7%)	1.7 ± 0.8 (0.6–3.3)	
	5+ servings	1 (1.4%)	0.7 ± 0 (n/a)	
Protein 5.5 servings (5.5 oz. eq.)			**Group total** **6.3 ± 3.3 (95% CI:5,5)**	0.059
	0 servings	1 (1.4%)	5.2 ± 0 (n/a)	0.000
	1–2 servings	24 (34.8%)	4.8 ± 2.6 (0.4–8.9)	
	3–4 servings	33 (47.8%)	6.3 ± 2.9 (2.6–12.8)	
	5+ servings	11 (15.9%)	9.7 ± 4.0 (3.9–16.8)	

Self-reported servings displayed as frequency (percent) by group and respective sub-group analysis from 3-day food record reported as Mean ± SD (Min-Max); (Min.-Max.) = Sub-group Range: minimum value – maximum value; Rec. = Recommended Daily MyPlate Servings; SD = Standard Deviation; (95% CI) = 95% Confidence Interval: Lower Bound, Upper Bound; n/a = not applicable; eq. = equivalent; oz. = ounces; ^‡^ = Bonferroni-corrected *p*-value ≤ 0.01.

### 2.3 Statistical analysis

Data analyses were performed using the Statistical Package for Social Sciences 26. Descriptive statistics (means, standard deviations) for all participant characteristics and the average nutrient intakes were calculated. The primary analysis was 95% confidence intervals (CIs) for each nutrient (e.g., calcium, vitamin D) compared to the respective Daily Values (DV). The 95% CI was calculated for each nutrient from the average 3-day food record. A Bonferroni correction was applied to account for multiple comparisons, adjusting the significance threshold to 0.00156 (0.05/ 32 nutrients) to control for Type I errors and reduce the risk of false positives. Participants included both males and females with a large age variance, therefore the DV, based on DRI, was used as the comparison value (31). For the remainder of this text, the comparison reference will be abbreviated as DRI. Linoleic acid and Alpha-linolenic acid have no DV established, therefore, the DRI for females was used as the comparison since 79.7% of the sample was female. Secondary analysis was 95% CI for MyPlate food groups from the 3-day food records and a one-way ANOVA analysis to compare perceived daily MyPlate servings to recorded servings from the 3-day food record. A Bonferroni correction was applied to adjust for multiple comparisons among the recorded food groups, adjusting the significance threshold to *p* ≤ 0.01 (0.05/5). Frequency statistics were also used when appropriate to describe participant characteristics and nutrient intake.

## 3 Results

### 3.1 Participants

A total of 99 participants were identified and qualified based on inclusion and exclusion criteria. Ninety-nine participants finished the survey and were invited to participate in the 3-day food record. Eighty-two participants completed a food record, but only 70 participants completed all 3 days. One participant was removed due to incomplete survey responses. Therefore, *N* = 69 was included in the final analysis which included 14 males and 55 females. All participants were currently taking GLP-1RA with the majority taking Semaglutide (*n* = 37, 53.6%) and Tirzepatide (*n* = 23, 33.3%) followed by Dulaglutide (*n* = 8, 11.6%) and Liraglutide (*n* = 1, 1.4%). The duration of medication use among participants was as follows: 7.2% (*n* = 5) had been taking the medication for less than three months, 29% (*n* = 20) for four to six months, 24.6% (*n* = 17) for seven to twelve months, and 39.1% (*n* = 27) for over one year with 48% (*n* = 33) of the total sample planning to use a GLP-1RA indefinitely. Most of the participants identified as White/Caucasian (82.6%) which is consistent with lower rates of GLP-1RA usage in Asian, African American, and Hispanic individuals (30). Within this sample, 80% agreed GLP-1RA helped them lose more weight than traditional programs. Among participants, frequency statistics found the most common reported side effects were nausea (53.7%), diarrhea (27.8%), and fatigue (30.3%), while 19.3% of participants reported no side effects. Key participant descriptive and frequency statistics are reflected in Tables 3, 4.

**Table 3:** Descriptive demographics for participants.

Variable	Males (*n* = 14)	Females (*n* = 55)	Total mean ± SD (*N* = 69)	Total Min, Max	*p*-value
Age (years)	49.4 ± 13.9	49.6 ± 12.0	49.6 ± 12.3	22, 70	0.952
Height (cm)	176.8 ± 7.7	168.9 ± 6.8	166.7 ± 8.8	147.3, 190.5	0.000*
Starting Weight (kg)	123.0 ± 24.8	118.2 ± 29.9	119.1 ± 28.9	65.8, 227.7	0.591
Current Weight (kg)	111.4 ± 26.3	96.5 ± 25.9	99.6 ± 26.5	58.5, 172.4	0.073
Starting BMI (kg/m^2^)	39.6 ± 6.8	43.9 ± 11.0	43.0 ± 10.4	28.3, 83.5	0.205
Current BMI (kg/m^2^)	35.7 ± 7.5	35.9 ± 9.6	35.9 ± 9.1	23.1, 61.6	0.945

SD = Standard Deviation; Min = minimum; Max = Maximum; * *p* < 0.05 between males and females; BMI = body mass index; cm = centimeters; kg = kilograms; m = meters.

**Table 4:** Frequency statistics for participants.

Variable	Males (*n* = 12)	Females (*n* = 48)	Total (*n* = 60)
Current body mass index category			
Healthy weight (BMI 18.5 to 24.9)	0 (0%)	3 (6.25%)	3 (5.0%)
Overweight (BMI 25.0 to 29.9)	3 (25%)	13 (27.1%)	16 (26.7%)
Class 1 obesity (BMI 30.0 to 34.9)	5 (41.7%)	6 (12.5%)	11 (18.3%)
Class 2 obesity (BMI 35.0 to 39.9)	2 (16.7%)	10 (20.8%)	12 (20.0%)
Class 3 obesity (BMI 40 and higher)	3 (25%)	15 (31.3%)	18 (30.0%)

Variable	Males (*n* = 12)	Females (*n* = 48)	Total (*n* = 60)
Starting body mass index category			
Overweight (BMI 25.0 to 29.9)	1 (8.3%)	3 (6.25%)	4 (6.7%)
Class 1 obesity (BMI 30.0 to 34.9)	1 (8.3%)	6 (12.5%)	7 (11.7%)
Class 2 obesity (BMI 35.0 to 39.9)	5(41.7%)	10 (20.8%)	15 (25.0%)
Class 3 obesity (BMI 40 and higher)	5 (41.7%)	29 (60.4%)	34 (56.7%)

Variable	Males (*n* = 14)	Females (*n* = 55)	Total (*n* = 69)
Ethnicity			
Asian	1 (1.4%)	0 (0%)	1 (1.4%)
African American	3 (4.3%)	2 (2.9%)	5 (7.2%)
Hispanic/Latino	1 (1.4%)	3 (4.3%)	4 (5.8%)
White/Caucasian	8 (11.6%)	49 (71.0%)	57 (82.6%)

Variable	Males (*n* = 14)	Females (*n* = 55)	Total (*n* = 69)
Education level			
Some high school	0 (0.0%)	2 (2.9%)	2 (2.9%)
High school graduate or equivalent	3 (4.3%)	10 (14.5%)	13 (18.8%)
Trade or vocational school degree	2 (2.9%)	1 (1.4%)	3 (4.3%)
Some college	1 (1.4%)	16 (23.2%)	17 (24.6%)
Associate’s degree	3 (4.3%)	6 (8.7%)	9 (13.0%)
Bachelor’s degree	4 (5.8%)	15 (21.7%)	19 (27.5%)
Graduate or professional degree	1 (1.4%)	5 (7.2%)	6 (8.7%)

Displayed as count (percent) of the sample population.

### 3.2 Nutrient intake

**Figure 1:**
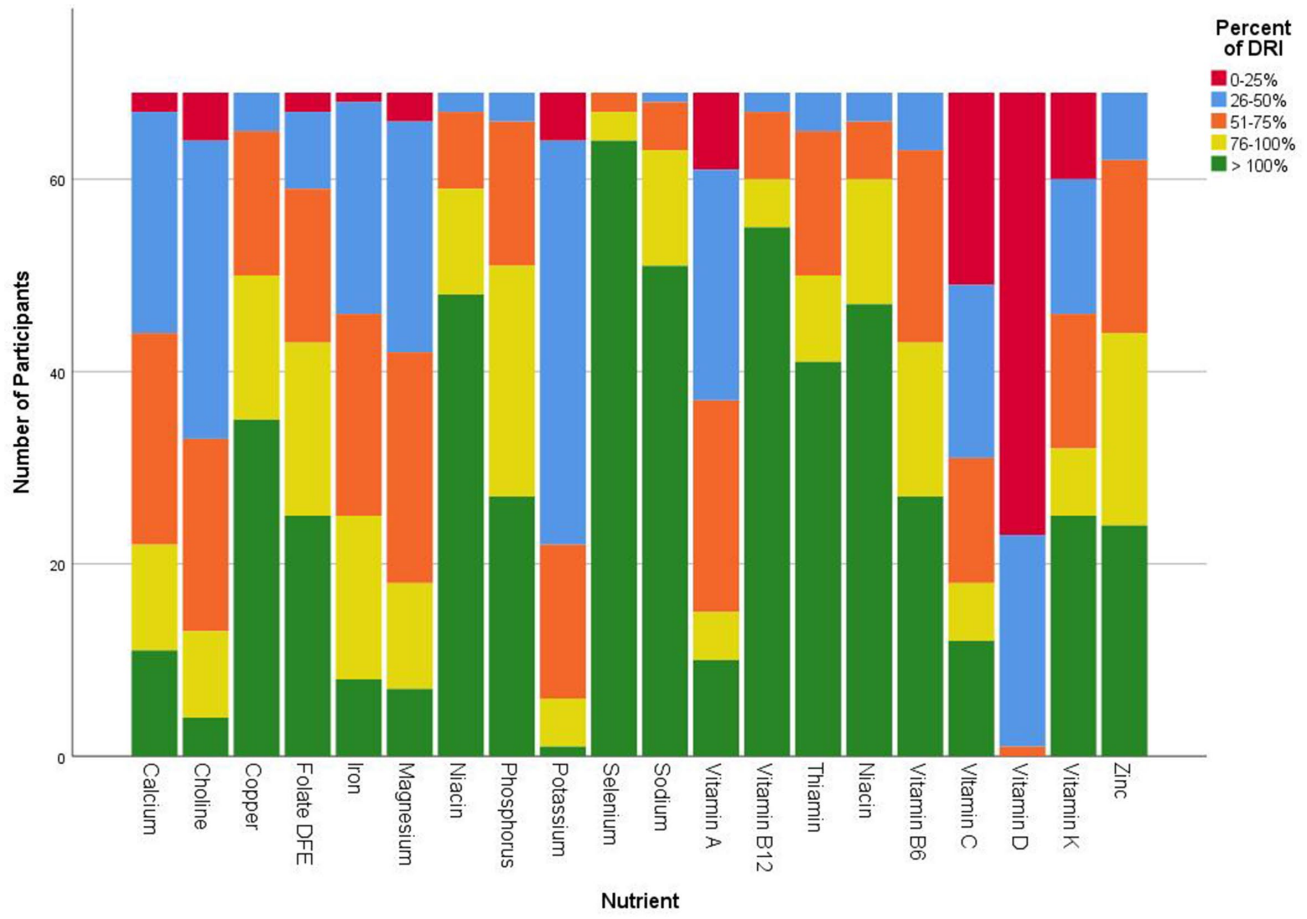
Frequency count categorized three-day average nutrient intake of GLP-1RA participants compared to dietary reference intake.

The average nutrient intake from the 3-day food records was compared to the DRI using 95% confidence interval in Table 1. The average calorie intake was 1,748 ± 651 (95% CI: 1,591, 1,905), with an average of 1,933 kcal/d for males and an average 1,700 kcal/d for females. Participants in this study consumed significantly above the DRI for fat (% of total calories), saturated fat, linoleic acid, alpha-linolenic acid, selenium, sodium, =, riboflavin, niacin, and vitamin B12 (*p* < 0.001). Carbohydrates (g/d and % total calories), fiber, calcium, iron, magnesium, potassium, vitamins A, C, D, E, and K, and choline were significantly under the DRI (*p* < 0.001). Protein intake (% total calories) was within the AMDR, however based on a g/kg/d, average protein intake (77.3 ± 29.2, 95% CI: 70, 84 g) was under consumed based on higher protein needs (1.2–2.0 g/kg calculated needs: 74–169 g/d). Comparison of nutrient intake frequencies revealed vitamin D, potassium, choline, magnesium, and iron had the largest deficits relative to the DRI standard, with 98.6, 98.6, 94.2, 89.9, and 88.4% of participants, respectively, falling below 100% of the recommended intake (see Figure 1). A one-way ANOVA found no significant difference in dietary intake for participants based on duration of GLP-1RA, ethnicity, or education levels (*p* > 0.05).

**Figure 2:**
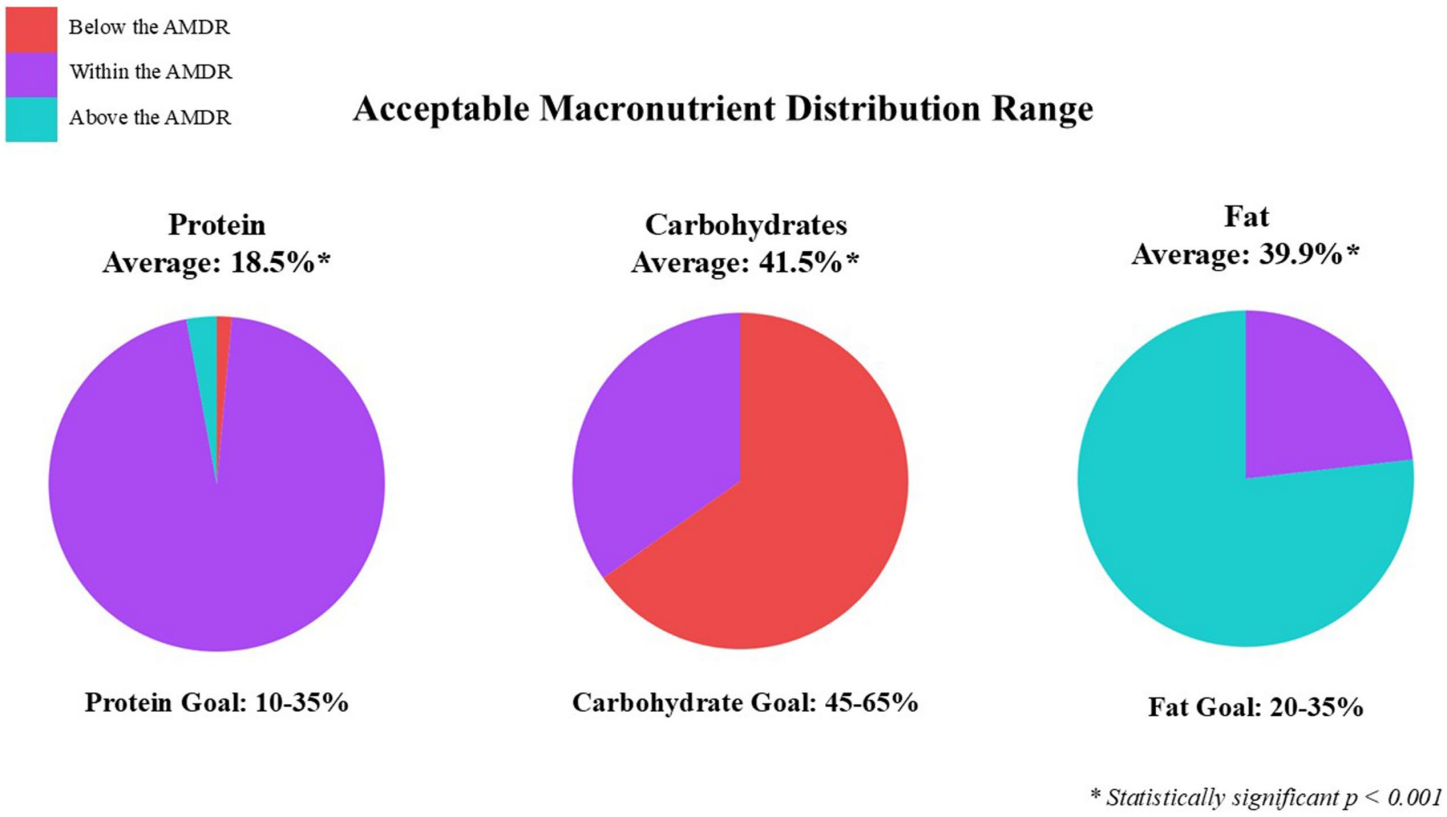
Categorized average three-day food record of GLP1-RA participants based on Acceptable Macronutrient Distribution Range (AMDR).

### 3.3 MyPlate

Participants completed a questionnaire self-reporting their typical daily servings from each food group (based on MyPlate) since beginning GLP-1RA. With MyPlate, one serving is defined as one cup equivalent (eq.) for fruits, vegetables, and dairy, and one ounce eq. for grains and protein. The recommended daily intake for the 1,800–2,000 calorie MyPlate guidelines include: 2 servings fruits, 2.5 servings vegetables, 3 servings dairy, 6 servings grains, and 5.5 servings protein. The 3-day food record was analyzed for the average daily servings of MyPlate food groups. Results of the self-reported food groups found most participants consume 1–2 servings of fruit (75.4%), vegetables (60.9%), grains (68.1%), and dairy (69.6%), and 3–4 servings of protein (47.8%) per day. The 95% CI from 3-day food records and Bonferroni correction revealed participants are significantly under-consuming the recommended servings of fruit, vegetables, grains, and dairy, based on MyPlate guidelines (*p* < 0.01; see Table 2). The 95% CI from 3-day food records for protein servings was not significant (*p* = 0.59). Overall, participants are not meeting the daily servings for fruit, vegetables, grains, or dairy food group recommendations based on self-reported servings or quantified food group servings from the 3-day food record. The 3-day food record descriptive statistics, self-reported frequency statistics, and one-way ANOVA are reported in Table 2.

## 4 Discussion

The purpose of this study was to assess nutrient intakes of GLP-1RA patients and compare averages to the DRI standards. This exploratory study was successful at analyzing specific nutrient intakes while using a GLP-1RA. Previous research on food intake with GLP-1RA participants did not analyze micronutrient levels (29). The primary finding of this study revealed that overall nutrient intake for this sample does not meet the DRI or MyPlate guidelines. Participants are consuming too many calories based on expert energy requirements, and too much sodium and saturated fat. Estimated energy requirements are personalized based on age, male/female, and activity level, however when analyzing group data this is not feasible. Based on expert recommendations, estimated energy requirements for individuals using a GLP-1RA are 1,200–1,500 kcal/d for females and 1,500–1,800 kcal/d for males (7). The average calorie intake was 1,748 kcal/d, which exceeds the average 1,500 kcal/d goal. The average calorie intake for males was 1,933, which is higher than the 1,500–1,800 kcal/d goal. The average calorie intake for females was 1,700 kcal/d, which is also higher than the recommended energy needs, showing excess calorie intake regardless of male or female. Similar to Silver et al., energy intake while using liraglutide exceeded the recommended calorie range and averaged 2,015 kcal/d after 14-weeks of treatment. Individual calorie needs should be calculated based on male/female needs, age, and activity level and adjusted throughout the weight loss journey to promote long-term weight management post GLP-1RA treatment (22). Following weight loss, metabolic adaptation can occur, reducing energy expenditure more than anticipated, which can hinder long-term success (32). Therefore, tailoring and adjusting caloric needs in a patient-centered approach throughout the weight loss process is crucial. Energy intake should also focus on appropriate macronutrient distribution and micronutrient quality.

Based on the AMDR, participants are consuming inadequate calories from carbohydrates (41.5%), excessive calories from fat (39.9%), and adequate calories from protein (18.5%) (see Figure 2). These findings are similar to previous studies where the percentage of total calories was 44.5, 36.4, and 17.1% carbohydrates, fat, and protein (22). Insufficient intake of fruit, vegetables, and dairy food groups further highlight the inadequate consumption of carbohydrates, where average intakes were 0.7, 1.2, and 1.4 cups, respectively. However, the average added sugar intake was relatively low at 11.5 grams, well below the DRI recommendation of 50 grams per day. This is likely influenced by a lower overall carbohydrate intake and a higher consumption of fatty foods reported. Interestingly, 72% of participants reported they are eating more fruits and vegetables since starting a GLP-1RA. It’s uncertain whether fruit and vegetable intake is higher than before starting a GLP-1RA, or if it’s due to a lack of awareness about portion sizes. For example, when surveyed on their fruit intake and provided examples of what a serving size was (e.g., one serving = 1 medium fruit, 1 cup of fresh fruit), participants self-reporting 3–4 servings of fruit did not consume that amount based on the food record. The sub-group analysis showed actual fruit intake averaged 1.2 servings per day. The ASA24 shows a visual example of portion sizes, which reflect more accurate reporting for the 3-day food record. This sample of participants consumed 0.2 servings more than the national median for fruit, and 0.4 servings less than the national median for vegetables based on MyPlate (33). In the U.S. only 25.3% of adults have heard of MyPlate and only 8.3% try to follow the recommendations, implying more education on well-balanced meals is needed (34). The lower intake of fruits and vegetables is evident by the lack of dietary fiber. This sample consumed on average 14.5 g/d, significantly less than the recommended 28 g/d. However, participants in this study are consuming less fiber than the national average intake of 16 g/d (35). The lack of fiber intake may also be tied to the quality of grains consumed by participants in this study, who over consumed refined grains (4.2 servings/d vs. recommended 3 servings) and under consumed whole grains (0.7 servings/d vs. recommended 3 servings). These results are consistent with national data which indicates that only 8% of adults meet whole grain recommendations where half of grain consumption should come from whole grains (36). A chief complaint for GLP-1RA users is upper gastrointestinal discomfort, such as constipation. Increasing fiber intake provides a nutritional strategy to help manage this common side effect (37).

This study also found participants over consumed fat (as a percentage of calories) based on the AMDR and saturated fat. Our findings indicate that participants consumed more calories from fat than from carbohydrates, with saturated fat intake averaging 6 g/d above the recommended daily saturated fat limits (38). Seventy percent of the U.S. general adult population exceeds the recommendations for saturated fat (38). Dietary fat naturally slows digestion and combining a higher-fat diet with GLP-1RA may further increase gastrointestinal discomfort. For this population, recommendations should emphasize shifting dietary fat intake and adjusting macronutrient distribution, specifically, reallocating calories to fiber-rich carbohydrates and protein. It is critical to highlight and address increased protein needs during weight loss and using a gram per kilogram approach is more optimal than AMDR. While our findings revealed an average 18.5% of calories were protein, this is inadequate for the GLP-1RA population. Several publications emphasize the importance of and critical need for high-protein diets during weight loss (14–16). To help preserve lean mass during hypocaloric diets, 1.2–2.0 g/kg of protein should be consumed daily (10). In this study, 75% of participants reported eating more protein since starting the GLP-1RA. However, only 43% consumed at least 1.2 g/kg of protein, 10% consumed at least 1.6 g/kg, and 5% consumed at least 2.0 g/kg, calculated based on adjusted body weight. This is alarming as maintaining muscle mass is a critical component of health and a diet composition focused on high protein intake during weight loss protects lean body mass (39–41). This study found notable gaps between actual protein intake and the recommended protein intake for adjusted body weight to help preserve lean mass during weight loss. The AMDR for protein was established to align with the protein intake necessary to meet the minimum RDA of 0.8 g/kg/d, with the upper limit reflecting the mathematical difference of the respective AMDR for carbohydrates and fat (42). This generalized framework may not sufficiently address the unique metabolic and nutritional considerations of individuals on GLP-1RA. This underscores the need for targeted research to refine these guidelines and further research is needed to confirm the optimal g/kg/d protein guidance while on GLP-1RA treatment. It is essential for RDNs to actively participate in the nutrition care process by calculating individualized protein needs rather than relying on AMDR as it does not adequately reflect higher protein requirements.

High-quality diets are necessary for GLP-1RA patients to attenuate unintended consequences of nutrient deficiencies and suboptimal protein intake. Adequate nutrient status is essential to support the body’s various functions and reduce the risk of chronic disease. This study found participants consumed enough B vitamins and too much sodium, however several other key nutrients were significantly under the DRI. Participants consumed 3,164 mg of sodium, which is 1.4 times the DRI. Within the sample, 73.9% of participants exceeded the DRI. Sodium intake is on par with the national average of 3,531 mg (38). Data from typical U.S. diets often fall below the DRI; in this sample, nutrient intake was not only below the DRI but also lower than the general U.S. population (38). For example, average calcium intake in this study was 862.9 mg, compared to 966 mg for the national average (38). Calcium, magnesium, and potassium are key nutrients highly correlated with cardiovascular health that were significantly under-consumed in this population (43). Only 1.4% of this sample met at least 100% of the DRI for Vitamin D. Vitamin D plays a key role in the absorption of calcium and magnesium. This study found an average of 4 mcg/d vitamin D, compared to the national average of 19 mcg/d from food and supplements, highlighting the significant need for Vitamin D-rich foods and supplementation for this population (44). Vitamin D is inversely associated with adiposity and more research is linking vitamin D deficiency and insulin resistance (45). Several other key nutrients were significantly below the daily recommendations and below the national averages. Recognizing the limitations of assessing nutrient intake for an entire group using standards based on a 2,000-calorie diet, this analysis still reveals substantial nutrient gaps in individuals on GLP-1RA. Despite lower caloric needs, established DRIs are determined by age and male or female, not energy requirements. Micronutrient deficiencies are often higher in people with obesity, and inadequate nutrient intake while using a GLP-1RA may be compounded by already low baseline levels. Continued poor nutrient intake failing to meet the DRI could lead to long-term detrimental outcomes. Extensive data exists on the potential adverse effects of micronutrient deficiencies; however, no studies have examined these outcomes specifically in the GLP-1RA population yet, as nutrition research in this area remains in early stages. This further highlights the need to address specific nutritional guidance for individuals on a GLP-1RA and the challenges of meeting DRI on a lower caloric diet. Additional research is needed to determine if individuals using a GLP-1RA have increased requirements for vitamins and minerals beyond the current DRIs.

As GLP-1RA therapies evolve, tailored nutritional guidance is essential to optimize health outcomes and prevent unintended consequences. In this study, only 51% of participants reported receiving information on how to manage potential side effects, 20% were referred to an RDN. More education, resources, and multi-disciplinary referrals are needed to better support a GLP-1RA patient. Across the spectrum of weight loss approaches, RDNs play a pivotal role in weight management. Studies show patients who receive ongoing nutrition counseling with RDN are more likely to maintain weight loss (46). Primary objectives for pharmaceutical weight loss should focus on optimizing diet composition and nutrient intake in addition to managing side effects. Overall, this study found participants consume too many calories, saturated fat, and sodium and not enough fiber, protein (g/kg/d), fruits, vegetables, and several vitamins and minerals (calcium, iron, magnesium, potassium, vitamin A, C, D, E, K, and choline). General guidance on higher protein intake calculated for g/kg must be emphasized and addressed to help preserve lean mass. Meal replacement shakes which are relatively low in calories and fat can provide a larger protein dose along with vitamins and minerals to help fill dietary gaps. This study is the first to explore nutrient intake in this population and further research is needed. However, preliminary nutritional guidance can be piloted in randomized clinical trials to assess outcomes.

The potential limitations of the current study include the following: (1) The convenience sample selected may not represent all individuals taking a GLP1-RA. However, this study provides initial insights on specific nutrient gaps to better tailor nutrition interventions for healthier weight loss outcomes. (2) This study compared average nutrient intake to the standard daily value limits based on a 2,000 calorie diet, thus not inclusive of lower calorie needs, activity levels, age or male and female-specific nutrient needs. (3) Demands based on exercise activity were not calculated, however hypo-caloric diets are necessary to elicit weight loss. Future studies are needed to determine the appropriate energy needs while using a GLP-1RA. (4) This study analyzed nutrients from food and beverage to align with the DRIs which are based on food sources, future studies should also analyze nutrient intake from dietary supplementation. (5) This study relied on self-reported data, assuming participants accurately reported dietary intakes. The ASA24 software provides strength in data collection for a 3-day food record as it provides visual examples of portion sizes, and probes for additional information to accurately gather nutrient intake. Nonetheless, all dietary assessment tools have their inherent weaknesses. For this study, the requirement to record their dietary intake on specific days potentially elicited behavior changes and introduced reporting biases. Future research should consider other validated methods for collecting dietary intake, such as biomarkers. (6) This study included participants using any type of GLP1-RA. Different medication forms such as semaglutide versus tirzepatide could result in different nutrient intakes. For example, side effects for tirzepatide are significantly higher than other GLP-1RA which may impact food intake (3). Given the exploratory nature of this study, we believe it represents initial steps to create more tailored nutritional guidance for GLP-1RA patients.

The use of GLP-1RA is rapidly growing, with global spending around $24 billion last year and some estimates propose global spending could reach $131 billion by 2028 (47). This growing demand highlights the increasing importance of developing evidence-based nutritional guidance for GLP-1RA. To our knowledge, no peer-reviewed data is available that describes the specific vitamin and mineral intake needed while concurrently using a GLP-1RA. Future large scale studies are needed to assess the replicability of average nutrient intakes. Additionally, studies should consider taking a 3-day food record before GLP-1RA begins, during treatment, and follow-up to better determine the changes of nutrient intake. Randomized-clinical trials are needed to assess various interventions combining GLP-1RA with controlled dietary interventions to evaluate health outcomes, such as nutrient status, muscle mass preservation, and overall quality of life. RDN plays a critical role in counseling this population to improve nutrient intake and support individuals on a weight loss journey.

## 5 Conclusion

This study aimed to assess nutrient intake while using a GLP-1RA compared to DRI. Overall, participants using a GLP-1RA consume too many calories, saturated fat, and sodium, adequate amounts of most B-vitamins, copper, phosphorus, selenium, and zinc and inadequate calcium, iron, magnesium, potassium, vitamin A, C, D, E, K, and choline. GLP-1RA participants did not meet the daily recommended MyPlate fruit, vegetables, grains, or dairy serving. Participants are also not meeting the g/kg/d protein needs to support lean mass during weight loss. Dietitians may use the results of this study as preliminary MNT guidance for this population. Future studies are needed to further develop guidance for this population and advance GLP-1RA-specific nutrition protocols.

## Data Availability

The datasets presented in this article are not readily available because we did not include this as part of the consent form. Requests to access the datasets should be directed to brittany-johnson@gnc-hq.com.
